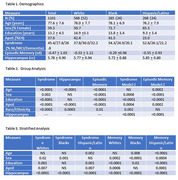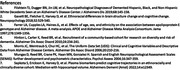# ApoE genotype and hippocampus have heterogenic influence on diagnosis and cognitive performance in the UCD ADRC longitudinal diversity cohort

**DOI:** 10.1002/alz.090892

**Published:** 2025-01-09

**Authors:** Eva Min, Sarah Tomaszewski Farias, David K Johnson, Lee‐Way Jin, Pauline Maillard, Evan Fletcher, Rachel A. Whitmer, Charles Decarli

**Affiliations:** ^1^ DePaul University, Chicago, IL USA; ^2^ University of California, Davis, Sacramento, CA USA; ^3^ University of California at Davis, Walnut Creek, CA USA; ^4^ University of California Davis Medical Center, Davis, CA USA; ^5^ Department of Neurology and Center for Neuroscience, University of California, Davis, CA USA; ^6^ UC Davis, Davis, CA USA; ^7^ University of California, Davis School of Medicine, Sacramento, CA USA; ^8^ University of California, Davis, CA USA

## Abstract

**Background:**

The UCD ADRC longitudinal diversity cohort consists of individuals from diverse backgrounds including White, Black African or Hispanic/Latino Americans that vary in cognitive ability from normal to dementia. Prior studies show differences in the pathological substrate of dementia in this group^1^ as well as the relationship between MRI measures and cognition^2^. Prior work also indicates differential influence of ApoE genotype on dementia by race^3^. For this study, we analyzed the influence of ApoE genotype and hippocampal volume on episodic memory performance and clinical syndrome of normal (NL), mild cognitive impairment (MCI) or dementia as a group and stratified by race/ethnicity.

**Method:**

Methods of participant recruitment and assessment for the UCD ADRC have been previously described^4^ Clinical syndrome was determined by a multispecialty group and coded according the UDS^5^. Episodic memory was measured by the SENAS^6^. Hippocampus was quantified using published methods^7^. Multivariate linear and logistic regressions were used to assess the relationships between ApoE4 genotype, diagnosis, cognition, and hippocampal volume for the entire group in addition to stratified analyses for each ethnoracial group. All analyses were adjusted for age, sex, and education (and race/ethnicity as needed).

**Result:**

Subject characteristics are summarized in Table 1. In brief, this is a cohort diverse in race/ethnicity, cognitive ability, and educational achievement.

For the entire group, odds ratios for ApoE4 prevalence were significantly higher comparing NL to MCI (1.7, p= 0.002), NL to Dementia (3.1, p <0.001) and MCI to dementia (1.7, p=0.004). ApoE4 (Table 2) was also significantly associated with smaller hippocampus (β=‐0.14, p<0.001)) and poorer memory (β=‐0.21, p<0.001). In models where hippocampus is included, both measures are associated with episodic memory and syndrome.

Different results were found for the stratified analysis where ApoE4 was not associated with syndrome or episodic memory among Blacks (Table 3). Hippocampal volume was also not associated with ApoE among Hispanic/Latinos.

**Conclusion:**

The relationship between ApoE genotype, hippocampus, syndrome, and memory performance is variable by ethnoracial background with only White participants showing associations of ApoE with both hippocampal volume and cognition. Further evaluation is needed to understand possible causes for these differences.